# The Health System Impact Fellowship: Perspectives From the Program Leads: Comment on "CIHR Health System Impact Fellows: Reflections on ‘Driving Change’ Within the Health System"

**DOI:** 10.15171/ijhpm.2019.59

**Published:** 2019-07-31

**Authors:** Meghan McMahon, Robyn Tamblyn

**Affiliations:** ^1^Canadian Institutes of Health Research, Institute of Health Services and Policy Research, Montreal, QC, Canada.; ^2^Institute of Health Policy, Management and Evaluation, University of Toronto, Toronto, ON, Canada.; ^3^Department of Medicine and Department of Epidemiology, Biostatistics and Occupational Health, McGill University, Montreal, QC, Canada.

**Keywords:** Health Services Research, Embedded Researcher, Learning Health Systems, Post-doctoral Training, Canada

## Abstract

As the Canadian Institutes of Health Research (CIHR) leads in designing and implementing the new Health System Impact (HSI) Fellowship program, we congratulate Sim et al for their thoughtful contribution to the nascent literature on embedded research, and for advancing our own learning about the HSI Fellowship experience. In our commentary, we describe the HSI Fellowship and its key components, discuss the factors that motivated and inspired the creation of the program, and highlight successes thus far.

## Introduction

As the Canadian Institutes of Health Research (CIHR) leads in designing and implementing this new Health System Impact (HSI) Fellowship program, we congratulate Sim et al^1^ for their thoughtful contribution to the nascent literature on embedded research, and for advancing our own learning about the HSI Fellowship experience. In our commentary, we describe the HSI Fellowship and its key components, discuss the factors that motivated and inspired the creation of the program, highlight successes thus far and suggest future directions for embedded fellowship models.

## Background: The Health System Impact Fellowship

In Canada, increased importance has been placed on modernizing doctoral programs to better prepare PhD graduates for stronger career readiness and greater impact in a wider variety of sectors and roles, within and beyond the academy.^2,3^ This is particularly relevant to health services and policy research (HSPR), where there is a need for skilled research capacity within healthcare organizations to enable evidence-informed improvement and system transformation within the context of a “learning health system.”^4,5^ For this reason, CIHR’s Institute of Health Services and Policy Research (IHSPR) created the HSI Fellowship program with three key objectives^6^:

- Support impact-oriented career paths and elevate the career readiness of PhD trainees and post-doctoral fellows by supporting experiential learning opportunities and the development of key professional competencies needed to succeed in healthcare organization environments;
- Expand and enrich the traditional doctoral and post-doctoral training environment by engaging health system and related organizations in preparing a cadre of promising doctoral trainees and post-doctoral fellows for successful careers; and
- Provide health system and related organizations with direct opportunities to realize and harness the benefits that research-trained individuals can bring to such organizations for improved decision-making.

The program aims to achieve these objectives by providing nationally-competitive fellowship awards that comprise a paid experiential learning opportunity within a health system organization where fellows dedicate the majority of their time towards a co-developed program of work designed to address a high-priority impact goal identified by the organization. In addition to experiential learning and an impact-oriented program of work, the HSI Fellowship has five distinct features:

1. Fellows are co-supervised by a health system leader within their “host partner organization” and by an academic with a university appointment, both of whom commit to mentoring the fellow and to fostering the fellow’s professional development and career preparedness.
2. Fellows spend the majority of their time embedded in their host partner organization but also have protected time for academic research (up to 30% protected time at the post-doctoral level). This is intended to ensure that fellows become immersed in the culture and operations of the organization and are able to make meaningful contributions to the organization’s impact goal, while also protecting time for academic research with their academic supervisor and providing a mechanism for fellows stay at the forefront of their fields and bring the latest research to bear on challenges faced by their host partner organizations.
3. In addition to their stipend, fellows receive a dedicated training allowance to support their professional development pursuits in areas that align with the HSI Fellowship program’s enriched core professional competencies (eg, leadership, negotiation). Fellows identify three of the 10 enriched core competencies to target for development at the outset of their fellowship, create a professional development plan and meet with their health system and academic supervisors to discuss their mentorship and support, and track their competency development over time using a standardized framework. The fellows’ regular self-assessments of their competency development (at baseline, three and 12 months) provides opportunities for the program leads to learn whether and how the HSI Fellowship is contributing to the fellows’ competency development and intervene with targeted support if needed.
The enriched core competency framework was developed in an iterative approach using qualitative methods and multiple sources of evidence – including a scoping review of the literature, key informant interviews with a purposeful national sample of health system employers and directors of HSPR PhD training programs, and quantitative data on HSPR PhD career trajectories – and was led by a 16-member Expert Working Group that was constructed to be broadly reflective of the key stakeholder groups involved in the HSPR training enterprise. The Expert Working Group reviewed the evidence, integrated it into options for HSPR training modernization, and validated the enriched core competencies at an invitational HSPR Training Modernization Symposium that brought together 100 leaders of HSPR training programs, health system organizations, research funders, trainees and international experts. Bornstein et al^7^ provide a comprehensive overview of the competencies and the rationale for the identification of those competencies.
4. Fellows and their health system and academic mentors are brought together in a national cohort whose annual in-person meeting and quarterly webinar sessions provide an important opportunity for training, learning and collaborations. The manuscript by Sim et al^1^ is an example of a collaboration that emerged from the relationships established at the National Cohort Retreat.
5. The application and peer review process are designed to reinforce the program’s emphasis on impact-oriented embedded research, co-supervision and leadership development. The application process requires that the applicant seek out a health system leader and academic supervisor committed to the fellowship objectives, co-develop an impact-oriented program of work proposal with the proposed supervisors, and confirm a host partner organization that will provide the required 30% funding contribution and a high-quality embedded training environment. The peer review focuses on four key criteria: professional and academic achievements of the applicant; quality of the host partner organization’s and academic institution’s training environment, supervision and mentorship; potential impact and feasibility of the program of work proposal; and potential value-add to the applicant and the host partner organization.

## Program Motivation: The Case for Embedded Fellowships

The HSI Fellowship was created in the context of three key motivating factors. First, there is a glaring disconnect between the career trajectories for today’s PhD graduates – which are diverse, non-linear and which often involve multiple non-academic sectors – and existing PhD training programs that remain predominantly geared towards academic careers. Recent data from Canada indicate that only about 19%^8^ to 30%^9^ of graduates (in all disciplines) find employment in tenure-track professor positions. Although we found in a recent career trajectory study that these percentages are higher in the field of HSPR – 42% of individuals that graduated from a PhD program in HSPR over the last 20 years were employed as a university professor in 2016 – we also found that employment trends have changed over time and that recent HSPR PhD graduates are less likely than past graduates to work in academia and more likely to work in a variety of sectors, including the public, private, not-for-profit, healthcare delivery and independent sectors.^10^ To ensure that PhD graduates are prepared to contribute fully and make an impact within these diverse sectors and roles, university-based doctoral training programs must evolve to keep pace with employment trends. Graduate training that centres on traditional academic skills alone is not enough in today’s knowledge economy.^11^ To make meaningful and impactful health and societal contributions throughout their careers, trainees need to complement their academic skills with professional competencies – such as leadership, management, interdisciplinary collaboration, communication, and change management – and have opportunities to develop and apply their skills within complex, dynamic “real world” settings. However, a 2016 survey of the heads of university-based HSPR doctoral training programs in Canada revealed that the vast majority of training programs do not explicitly include professional competencies or experiential learning opportunities in their core curriculum.^10^ It is therefore not surprising to learn in the consultations that informed the HSI Fellowship that while many PhD students aspire to administrative and management roles within the health system or hybrid careers that span academic and health system settings, many graduates who moved into non-academic careers feel they were underprepared to do so and under-utilized in such roles.^7^ These findings appear to be in line with doctoral graduate experience overall.^12^ Thus, the HSI Fellowship was created as one mechanism to address the training-employment disconnect and to equip PhD trainees and post-doctoral fellows with the skills, experiences and networks to pursue and succeed in a diverse array of employment sectors and roles.

The second motivating factor was demand from health system organizations for research talent in their teams. The complexity and multitude of challenges facing governments and health system organizations requires research and analytic expertise to experiment with innovations, evaluate and learn from past approaches and ensure policies and programs are informed by the best available evidence.^4,5^ A diversity of health system organizations in Canada – including hospitals, ministries of health, health authorities, health charities and others – now have embedded research, business intelligence and/or quality improvement units. Many forward-looking organizations are making strides to use data to inform continuous improvements and are employing researchers in a variety of roles. Within this evolving landscape, the potential contribution of well-prepared PhD graduates to inform health policy and system transformation is considerable and, we learned in the work leading up to the creation of the HSI Fellowship, valued by many different kinds of health system organizations. We found that health system organizations were keen to partner in efforts to modernize HSPR training and engage with research funders and universities to train a new cadre of PhD graduates. Thus, building on the theory and practice of integrated knowledge translation,^13^ the HSI Fellowship was created to build bridges between academia and the health system, to embed research talent and capacity directly within organizations, and to contribute to evidence-informed health system improvement.

The third motivation was the realization that there are pockets of innovation where change to modernize doctoral and post-doctoral training for impacts beyond the academic sector was already occurring. Efforts to rethink doctoral education are evident in the humanities^[1]^ and basic sciences^[2]^, and a growing number of universities in Canada and other countries are spearheading novel approaches to training for broader career readiness and enhanced societal contribution^[3]^. Three programs in particular inspired our thinking about the HSI Fellowship program design.

Mitacs, a not-for-profit Canadian organization that builds partnerships between academia and industry, offers a research internship award designed to increase deployment of PhD trainees and post-doctoral fellows in the private sector. Its “Accelerate” and “Elevate” programs provide successful PhD students and post-doctoral fellows with a paid award to partner with a company, undertake a collaborative research-based project, and develop career skills through on-the-job training (OJT) and a specialized professional development program.^14^

In the United States, AcademyHealth’s Delivery System Science Fellowship provides a paid one-year fellowship for “highly qualified, doctorally-prepared individuals interested in enhancing and applying their analytic skills to relevant and timely research topics in a delivery system setting.”^15^ Fellows are embedded within delivery organizations and contribute their research and analytic talents to complex, real-time challenges those organizations are facing. There is such demand for fellows that 16 delivery organizations are now involved and pay the full cost of the fellow’s salary.^16^

Also in the United States, the Pardee RAND Graduate School features OJT as an integral aspect of its doctoral degree requirements and also includes professional skills among the core competencies taught to all students. The program is founded on a “learning by doing” philosophy and recognizes that “classroom exercises alone do not create a superb policy analyst. Also essential is hands-on experience at dealing with real-world problems of direct concern to decision-makers.”^17^ A robust monitoring and evaluation framework is in place to continuously measure OJT learning and inform continuous program improvement.^17^

Other countries have also increased their investment in training more individuals at the doctoral level specifically to improve competitiveness, innovation and industry performance. The European Commission’s Marie Skłodowska-Curie Actions is an example of a large-scale funding program focused explicitly on aligning research training with career development in business sectors, involvement of industry in doctoral training, and on developing researchers’ innovation skills for deployment in industry.^18^

These different programs share the goals of preparing a cadre of highly educated graduates to meet increasing demand in health system organizations by exposing doctoral trainees and post-doctoral fellows to careers outside of traditional academic settings, preparing them for success and impact by providing them with experiential and professional skills development learning opportunities and senior mentorship. They also show that it is possible to integrate broader sets of competencies into PhD education while maintaining the rigour and depth of scholarly preparation, and to increase the capacity of PhD graduates to enhance industry and organizational performance.

## Early Indications of Success

There is much to learn from the experience and outcomes of the initial cohorts of fellows and their health system and academic supervisors. In the program’s first two years, 62 health system organizations and 23 academic training programs across Canada have co-trained 95 HSI Fellows. The number and diversity of organizations involved – including hospitals, federal and provincial government agencies, health charities, consulting firms, health professional associations and non-university research institutes – are encouraging indications of the demand for PhD skills within Canada’s health systems. The number and diversity of impact projects that fellows are engaged in– including, as illustrative examples, informing the development of a province-wide population health policy framework; testing new models of care to support healthy aging; evaluating health system-mediated interventions that target the upstream determinants of health; optimizing home, community-based and palliative care; and evaluating the impact of e-health innovations on patient safety (the full list of projects as well as fellows’ profiles are available on the CIHR website^19,20^) – provides a glimpse into the breadth of challenges confronting our health system and also illustrates that HSPR PhD skills are perceived as relevant and valuable across a range of complex issues.

In addition to demand from health system organizations for PhD talent, the increasing number of applications from PhD trainees and post-doctoral fellows in the program’s first two years is an indication that experiential learning opportunities at the coal face of health policy and service delivery, paired with co-mentorship from health system and academic leaders and a focus professional skills development are valued by today’s PhD trainees. Our early efforts to systematically study and learn from the experiences of the first cohorts of fellows and supervisors and about the contributions they are making have revealed that the program is providing an opportunity for fellows to develop core competencies that are not currently emphasized in HSPR doctoral curriculum^21^; that the dual health system-academic training and mentorship approach has demonstrated positive impacts to fellows, supervisors and host partner organizations^22^; that fellows and their health system and academic supervisors agree that a key contribution the fellows bring is their research and analytic skills and expertise^23^; and that the first cohort of fellows are transitioning into exciting careers in the academic, public (government) and not-for-profit sectors and in primarily research-related roles that draw on their PhD skills.

## Conclusion

There is an increasing need to modernize traditional doctoral and post-doctoral training in all academic domains. The recognized value of embedding advanced research capacity into health delivery systems has catalyzed the design and successful implementation of an innovative partnership program to enhance traditional academic training.

## Ethical issues

Not applicable.

## Competing interests

Authors declare that they have no competing interests.

## Authors’ contributions

Both authors conceptualized the manuscript. MM drafted the initial version and RT provided a critical revision. Both authors read and approved the final manuscript.

## Authors’ affiliations

^1^Canadian Institutes of Health Research, Institute of Health Services and Policy Research, Montreal, QC, Canada.^2^Institute of Health Policy, Management and Evaluation, University of Toronto, Toronto, ON, Canada.^3^Department of Medicine and Department of Epidemiology, Biostatistics and Occupational Health, McGill University, Montreal, QC, Canada.

## Endnotes


[1] See for example The Future of the PhD in Humanities (http://carleton.ca/phdhums/future-of-the-phd-in-the-humanities/).


[2] Example the University of Toronto’s Department of Biochemistry’s Professional Development program.


[3] Select examples include the University of British Columbia’s Public Scholars Initiative and the Pardee RAND Graduate School.
